# Evaluation of magnetic resonance imaging and clinical outcome after tissue-engineered cartilage implantation: prospective 6-year follow-up study

**DOI:** 10.1007/s00776-012-0231-y

**Published:** 2012-05-12

**Authors:** Kobun Takazawa, Nobuo Adachi, Masataka Deie, Goki Kamei, Yuji Uchio, Junji Iwasa, Nobuyuki Kumahashi, Taku Tadenuma, Suguru Kuwata, Kazunori Yasuda, Harukazu Tohyama, Akio Minami, Takeshi Muneta, Shigeo Takahashi, Mitsuo Ochi

**Affiliations:** 1 Department of Orthopaedic Surgery, Hiroshima University, 1-2-3 Kasumi, Minami-ku, Hiroshima, 734-8551 Japan; 2 Department of Orthopaedic Surgery, Shimane University School of Medicine, Izumo, Japan; 3 Department of Sports Medicine, Hokkaido University School of Medicine, Sapporo, Japan; 4 Department of Orthopaedic Surgery, Hokkaido University School of Medicine, Sapporo, Japan; 5 Department of Orthopaedic Surgery, Tokyo Medical and Dental University, Tokyo, Japan; 6 Department of Orthopaedic Surgery, Mitsubishi Nagoya Hospital, Nagoya, Japan

## Abstract

**Background:**

Autologous chondrocyte implantation (ACI) is an important procedure when repairing cartilage defects of the knee. We previously reported several basic studies on tissue-engineered cartilage, and conducted a multicenter clinical study in 2009. In this clinical study, we evaluated the patients’ clinical scores and MRI findings before and after tissue-engineered cartilage implantation, and compared the data obtained at 1 year and approximately 6 years post-implantation.

**Methods:**

Fourteen patients who underwent implantation of tissue-engineered cartilage to repair cartilage defects of the knee were evaluated. Tissue-engineered cartilage was produced by culturing autologous chondrocytes three dimensionally in atelocollagen gel. The patients were evaluated clinically using the Lysholm score, and the original knee-function score at pre-implantation and at 1 year and approximately 6 years post-implantation. MRI scans were obtained at the same observation periods. A modified magnetic resonance observation of cartilage repair tissue (MOCART) system was used to quantify clinical efficacy based on the MRI findings.

**Results:**

In approximately 6 years of follow-up, none of the 14 patients reported any subjective symptoms of concern. The mean Lysholm score and the original knee-function score (63.0 ± 10.1, 59.9 ± 5.7) significantly improved at 1 year after implantation (86.4 ± 11.8, 94.1 ± 9.2), and were maintained until 6 years after implantation (89.8 ± 6.2, 89.9 ± 11.2), although some patients showed deterioration of Lysholm and original knee scores between 1 year post-implantation and the final follow-up. The mean MOCART score was 13.2 ± 12.0 pre-implantation, and 62.5 ± 24.7 at 1 year and 70.7 ± 22.7 at approximately 6 years post-implantation. The MOCART scores at 1 year and 6 years were significantly higher than the pre-implantation score, but there was no significant difference between the scores at 1 and 6 years, indicating that the MRI results at 1 year after implantation were maintained for the next 5 years.

**Conclusions:**

The clinical scores and MRI findings after implantation of tissue-engineered cartilage were improved at 1 year after implantation and were maintained until 6 years after implantation.

## Introduction

There have been numerous reports on the use of cultured cells to treat cartilage injuries of the knee. One of the most prominent reports, by Brittberg et al. [1], is on autologous chondrocyte implantation (ACI). In that work, the authors used monolayer culture to increase the number of chondrocytes from cartilage harvested from healthy non-weight-bearing sites, and then transplanted these cells to repair articular cartilage defects after covering the defects with a periosteal flap with stitches. Although a number of concerns relating to this conventional ACI have been highlighted in subsequent reports, or adverse events after implantation were reported [2–5], it appears that this new surgical technique has now become an established procedure, and excellent results have been reported [6–9]. Recently, the autologous periosteum was replaced by a collagen membrane used as a covering material to avoid some adverse events and the invasion of healthy tissue [10]. Moreover, options for ACI are already commercially available, and this treatment approach is now considered to have become routine.

Through an assessment of normal chondrocytes cultured three-dimensionally in agarose gel, Benya and Shaffer [11] found that these cells maintained a cartilage-organizing potential similar to that in living organisms without dedifferentiation, in contrast to the results obtained in a monolayer culture. In addition to showing that cultured chondrocytes in atelocollagen gel maintained their cartilage-organizing potential, we have demonstrated the usefulness of this cultured cartilage in animal experiments [12, 13]. Based on these studies, we modified the conventional ACI approach using isolated cultured chondrocytes to devise a method for implanting tissue-engineered cartilage using three-dimensional culture in atelocollagen gel [14]. In short, we performed transplant procedures in 28 knees and conducted follow-ups for at least 25 months, reporting excellent results in 26 knees. Using this technique, we sought to address the potential disadvantages of conventional ACI, such as dedifferentiation in monolayer culture and leakage of chondrocytes. Recently, many types of biodegradable materials have been used as scaffolding to make three-dimensional cultured cartilage [15–17].

Approaches to the clinical evaluation of therapies for knee injury such as ACI include subjective evaluations by patients using various clinical scores. Two of these, the knee injury and osteoarthritis outcome score (KOOS) [18] and the Lysholm knee score (LKS) [19], are important indicators for elucidating clinical usefulness. Meanwhile, objective evaluation methods include arthroscopically-guided diagnostic imaging and magnetic resonance imaging (MRI) evaluation. The greatest advantage of MRI evaluation is that it permits noninvasive imaging and evaluation, and a comparison of MRI changes over time is useful. However, since it requires specialized equipment and is burdensome for certain patients, and moreover, since routine evaluation indicators are not especially well established, few reports have been published on MRI changes in the long-term follow-up of patients who have undergone ACI.

With the aim of further establishing the usefulness of tissue-engineered cartilage using atelocollagen, a multicenter study was conducted in 2009 by a group that included the authors, in which cartilage defects of the knee in 27 patients were treated [20]. This was conducted as a sponsor-initiated clinical study on behalf of a company which focuses solely on regenerative medicine. In short, the study was conducted at six orthopaedic centers that specialize in the treatment of knees. Evaluations of variables such as clinical symptoms and arthroscopic findings revealed improvement according to our original knee-function score, and arthroscopic examination yielded evaluations of normal or nearly normal results in 92 % of knees, demonstrating the usefulness of cultured cartilage grown in three-dimensional culture using atelocollagen.

In contrast to the number of reports on the long-term follow-up of patients with conventional ACI [6, 7, 21], there are few reports on the long-term follow-up of the fate of three-dimensionally cultured chondrocytes. Furthermore, for the reasons presented above, the literature is silent on longitudinal studies of MRI evaluations. Therefore, for patients who were part of the multicenter study and were then followed up at our centers, we describe in this report a comprehensive investigation of changes over the follow-up duration in these patients’ clinical symptoms and MRI findings for a mean duration of at least 6.2 years after implantation.

## Materials and methods

### Subjects

The subjects of this investigation were drawn from the 31 patients described in the report published by Tohyama et al*.* [20]. From the 30 patients who were included in the efficacy evaluation set, we recruited those who were available for a 5-year follow-up at two medical institutions in Japan.

The inclusion criteria in the clinical study conducted by Tohyama et al. were as follows: adults aged 20 years or older; patients with full-thickness defects of cartilage in knees caused by trauma, or osteochondritis dissecans (OCD) or osteoarthritis (OA); and patients who had either failed to respond to conventional methods or for whom it was judged that a benefit could not be anticipated. Similarly, the study was conducted with appropriately-selected exclusion criteria such as a history of rheumatoid arthritis and other systemic diseases, or of malignant tumor. Patients were also subjected to intradermal testing to establish that they were not allergic to atelocollagen gel.

From the 30 patients evaluated for efficacy in the above multicenter study in six medical institutions, the present follow-up research was conducted for 18 patients at two centers. We evaluated a total of 14 patients, excluding those who were unable to visit the centers for personal reasons. The mean age of these evaluated patients was 33.1 years (21–52), six being male and eight female. The causes of the osteochondral defects were trauma (11 knees) and osteoarthritis (3 knees), and the mean (±SD) lesion size was 3.4 (±2.7) cm (Table 1). Two patients underwent simultaneous surgical procedures, with concomitant reconstruction of the medial patellofemoral ligament (case nos. 3, 10). Details of the patients are summarized in Table 1.

**Table 1:** Details of the 14 knee with cartilage defects treated with cultured cartilage transplantation

Case	Gender	Age (years)	Height (cm)	Body weight (kg)	Disease	Side	Site of lesion	Size of lesion (cm^2^)	Outerbridge grade	Number of previous operations	Duration of symptoms (months)	Follow-up; EP II (years)
1	F	36	152	45.0	Trauma	L	MFC	1.3	III	0	11	6.7
2	M	26	173	74.0	Trauma	L	LFC	2.4	III	1	24	6.6
3^a^	F	21	152	42.8	Trauma	L	Patella	2.0	IV	0	60	6.4
4	M	45	169	87.0	Trauma	L	MFC	2.9	IV	1	12	6.3
5	M	30	169	52.2	Trauma	L	MFC	2.0	IV	0	3	6.1
6	M	42	167	74.6	OA	L	MFC	2.4	III	0	24	6.1
7	M	23	177	75.0	Trauma	R	MFC	1.6	IV	1	3	6
8	F	22	168	62.0	Trauma	R	MFC	3.8	IV	0	3	6.6
9	F	47	160	65.0	OA	R	Patellar fossa	2.8	IV	1	9	6.3
							Patella	1.3				
							MFC	0.6				
10^a^	F	21	151	45.0	Trauma	L	LFC	2.0	IV	0	3	6.3
11	M	40	171	91.0	Trauma	R	LFC	4.9	IV	1	36	6
12	F	37	160	59.0	Trauma	L	Patella	4.1	IV	0	3	5.9
							Patellar fossa	2.9				
13	F	52	162	82.0	OA	L	MFC	11.3	IV	0	24	5.7
14	F	22	171	44.0	Trauma	L	Patella	4.2	IV	1	60	5.7

*OA* osteoarthritis, *EP II* the final evaluation time, *LFC* lateral femoral condyle, *MFC* medial femoral condyle

^a^Reconstruction of medial patellofemoral ligament was performed simultaneously during the operation

### Preparation of tissue-engineered cartilage

Tissue-engineered cartilage was prepared according to the method described by Ochi et al. In short, approximately 300 mg of normal cartilage was harvested from an unloaded site under arthroscopic guidance from each individual patient and delivered to a facility belonging to Japan Tissue Engineering Co., Ltd. (Gamagori, Japan). All tissue-engineered cartilage used in this multicenter study was prepared at this facility. After arrival at the facility, the patient’s cartilage tissue was processed with collagenase (type XI; Sigma-Aldrich, St. Louis, MO, USA) to produce a suspension of isolated chondrocytes. The medium used for seeding was Dulbecco’s Modified Eagle Medium (DMEM; GIBCO Invitrogen, Carlsbad, CA, USA) supplemented with 10 % fetal bovine serum (FBS) (JRH Biosciences, St. Lenexa, KS, USA) and 20 mg of HEPES buffer (GIBCO Invitrogen).

To perform the three-dimensional culture in atelocollagen, the suspension of isolated chondrocytes in the above medium and atelocollagen (3 % type I collagen; Koken, Tokyo, Japan) were mixed in a 1:4 ratio and then stirred thoroughly to produce a uniform mixture. The mixture of thoroughly-dispersed cells and atelocollagen was added to culture dishes using a special-purpose seeding ring, and the dishes were heated at 37 °C for 1 h to harden the gel.

The medium used for cell culture was DMEM supplemented with 10 % fetal bovine serum (FBS), 50 μg/ml l-ascorbic acid phosphate magnesium salt (Nikko Chemicals, Tokyo, Japan), 50 μg/ml gentamicin sulfate (Schering-Plough, Munich, Germany), 0.25 μg/ml amphotericin B (Bristol-Myers Squibb, New York, NY, USA), and HEPES buffer. FBS was selected in accordance with the requirements of the Standards for Biological Ingredients (notification no. 210 of the Japanese Ministry of Health, Labor and Welfare).

The tissue-engineered cartilage was incubated in an atmosphere of 5 % carbon dioxide and 95 % air at 37 °C. The culture medium, which was well qualified by means such as a sterility test, was changed every 3–4 days. As the cell culture progressed, the collagen became opaque and acquired a certain level of hardness. Furthermore, cell outgrowths were observed from the locations where the gel was attached to the dish, and cells also became visible on the dish surface.

### Implantation of cultured cartilage

Chondrocytes were three-dimensionally cultured in atelocollagen gel for 28 days. The atelocollagen gel containing these chondrocytes was used as the tissue-engineered cartilage for grafting. Before being shipped as tissue-engineered cartilage from the culture facility, a suite of quality tests was performed. Briefly, the results of these pre-shipment quality tests consisted of a negative bacterial cultivation test of the medium, a negative membrane filter sterility test, a negative* Mycoplasma* screening test using polymerase chain reaction (PCR), a negative endotoxin test, the number of viable cells (by microscopic examination to determine cell number) and the viability (with a hemocytometer and Trypan blue staining), cellular outgrowth from the tissue-engineered cartilage, glycosaminoglycan content, and bovine serum albumin content.

A medial or lateral parapatellar arthrotomy was carried out under tourniquet control. The chondral lesion was debrided as far as the normal surrounding cartilage and until subchondral bone was visible. The defect was covered with a sutured periosteal flap taken from the proximal medial tibia. The flap was shaped and sutured to the surrounding rim of normal cartilage with interrupted 5-0 nylon. After suturing half of the border of the flap, the tissue-engineered cartilage was placed in the defect, and the remaining border of the flap was sutured. The joint capsule, retinaculum, and skin were sutured in separate layers. The knee was supported by a lightweight brace. Two weeks after transplantation, continuous passive movement of the joint was begun. Partial weight-bearing was introduced 3 weeks after surgery, and was gradually increased to full weight-bearing with muscle training during the first 8 weeks after surgery.

### Multicenter study procedures

As described above, 14 patients treated at two medical institutions were selected as the subjects of this study. Before the study commenced, the ethics committees of each university reviewed and approved the ethical validity of the study. Written informed consent was obtained from all patients, and the rights of the patients were protected. All patients agreed that any results of this study would be published. Patients selected as subjects were asked to visit the appropriate hospital, and MRI scans were obtained after interviews about their clinical findings at that time. Further details of the procedures are provided below.

### Evaluation by clinical outcome

To produce a score for clinical symptoms, the interview included questions about symptoms such as motion pain, rest pain, and knee motion, and the Lysholm–Gillquist scores [19] were determined from the responses. These scores were also converted to a numerical value using our original knee-function score [20], which is optimized for evaluating the implantation of tissue-engineered cartilage (Table 2).

**Table 2:** Descriptions of the Lysholm knee score and the original knee function score

Lysholm knee score		Original knee-function score	
Description	Score	Description	Score
Limp		Knee motion pain	
None	5	No motion pain	50
Slight or periodic	3	Mild motion pain (rare, relieved)	35
Severe and constant	0	Moderate motion pain (frequent, limiting)	20
Support		Severe motion pain (constant, not relieved)	0
None	5	Rest knee pain	
Stick or crutch needed	2	No rest pain	25
Weight-bearing impossible	0	Mild rest pain (rare, relieved)	15
Locking		Moderate or severe rest pain (frequent or constant)	0
None	15	Range of knee motion	
None, but catching sensation present	10	No loss of motion	25
Occasional	6	Mild loss of motion (total are ≥90°)	16
Frequent	2	Moderate loss of motion (total are <90°)	8
At examination	0	Ankylosis	0
Stairs		Total	100
No problem	10		
Slight problem	6		
One step at a time	2		
Impossible	0		
Instability			
Never	25		
Rarely during athletic activities	20		
Frequently during athletic activities	15		
Occasionally during daily activities	10		
Often during daily activities	5		
Every step	0		
Pain			
None	25		
Inconstant and slight during strenuous activities	20		
Marked during or after walking more than 2 km	10		
Marked during or after walking less than 2 km	5		
Constant	0		
Swelling			
None	10		
After strenuous activities	5		
After ordinary activities	2		
Constant	0		
Squatting			
No problem	5		
Slight problem	4		
Not beyond 90° of knee flexion	2		
Impossible	0		
Total	100		

An evaluation of the clinical course was made by comparing the scores obtained pre-implantation (pre-operation period; PP) and at 1 year post-implantation (evaluation period I; EP I). At each final hospital visit, patients were asked about their clinical symptoms at the time of evaluation [evaluation period II; EP II, 6.2 (5.7~6.7) years post-implantation].

We selected our original knee function score because, in contrast to the Lysholm knee score (LKS), which includes items relevant to evaluating therapeutic efficacy after ligament reconstruction, such as limping and knee stability, our score minimizes the effects of such factors and considers only the effects of cartilage.

We not only inquired into the level of knee loading in daily activities, but also ascertained whether patients had undergone additional surgical interventions since implantation.

### Evaluation by MRI

Magnetic resonance imaging was done on a 3.0 T magnet system (Signa EXCITE HD 3T, Signa HDx 3T; GE Healthcare, Little Chalfont, UK). During imaging, the knee was flexed slightly and scans were acquired under proton density-weighted conditions. Coronal and sagittal scans were acquired for the femoral condyle, and axial and sagittal scans for the patellofemoral joint surface.

After imaging, a modified version of the magnetic resonance observation of cartilage repair tissue (MOCART) system [22, 23] was used to score the extent of cartilage formation at the transplanted site (Table 3). Evaluations were done by an orthopaedic surgeon who was involved in clinical and basic research into cultured cartilage, but was not involved in treating the patients of this study. Multiple defects were evaluated individually, and the worst score was adopted as the patient MOCART score. The modifications were as follows: under the original MOCART method, images were obtained using fast spin echo and 3D-gradient echo-FSE sequences, while we employed a single imaging sequence and scored a maximum of 30 points for items relevant to the signal intensity of the image (in the original method, a total of 30 points, comprising 15 points per item, was used). For the reconstructed region of cartilage visualized on the MRI scans, the height of the formed cartilage, the integration to the border zone, the surface of the repaired tissue, and other variables were evaluated on a score with a maximum score of 100. For these items, we compared scores obtained at three times: pre-implantation (PP), at 1 year post-implantation (EP I), and at the final evaluation time (EP II).

**Table 3:** Description of the modified MOCART score

Variable	Class	Score
Degree of defect repair and defect filling	Complete (on a level with adjacent cartilage)	20
	Hypertrophy (over the level of the adjacent cartilage)	15
	Incomplete (under the level of the adjacent cartilage: underfilling)	
	>50 % of the adjacent cartilage	10
	<50 % of the adjacent cartilage	5
	Subchondral bone exposed (complete delamination or dislocation and/or loose body)	0
Integration to border zone	Complete (complete integration with adjacent cartilage)	15
	Incomplete (incomplete integration with adjacent cartilage), demarcating border visible (split-like)	10
	Defect visible	
	<50 % of the length of the repair tissue	5
	>50 % of the length of the repair tissue	0
Surface of the repair tissue	Surface intact (lamina splendens intact)	10
	Surface damaged (fibrillations, fissures, and ulcerations)	
	<50 % of repair tissue depth	5
	>50 % of repair tissue depth or total degeneration	0
Structure of the repair tissue	Homogeneous	5
	Inhomogeneous or cleft formation	0
Signal intensity of the repair tissue	Isointense	30
	Moderately hyperintense	10
	Markedly hyperintense	0
Subchondral lamina	Intact	5
	Not intact	0
Subchondral bone	Intact	5
	Edema, granulation tissue, cysts, sclerosis	0
Adhesions	No	5
	Yes	0
Effusion	No effusion	5
	Effusion	0
Maximum score		100

### Statistical analysis

To compare the LKS and other clinical scores, as well as the above MRI-related scores, the relationships between the respective scores obtained at PP, EP I, and EP II were statistically analyzed.

Multiple linear regression was used to statistically analyze the evaluations of the LKS and MRI scores, and Wilcoxon’s signed rank test was used to analyze the effects at each evaluation time (PP, EP I, EP II) for the MRI scores. Differences were considered significant at *p* < 0.05.

## Results

### Clinical course

At EP II (mean duration 6.2 years after implantation), none of the 14 patients reported any subjective symptoms of concern. However, 3 of these patients reported experiencing post-implantation pain and other transient subjective symptoms. Meanwhile, 8 patients reported engaging in daily activities that imposed an excessive load on the knee, such as sports or heavy physical labor, and denied any findings of concern at such times. Moreover, no patients had undergone any additional surgery up to 6 years post-implantation (Table 1). In the present study, there was no infection during the cell culture periods or after implantation, no deep thrombosis, neural or arterial involvement, nor ossification of the grafts.

### Evaluation of clinical scores (LKS and original knee-function score)

**Fig. 1:**
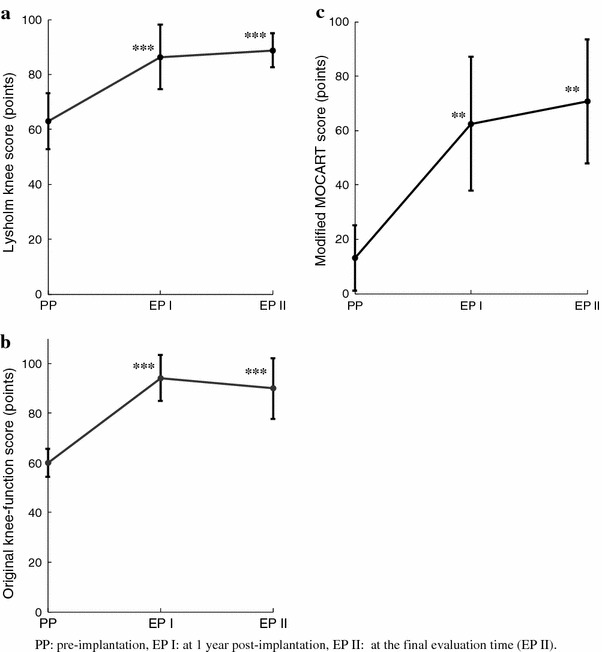
Mean improvements in clinical and MRI scores at PP, EP I, and EP II. **a** Lysholm knee score, **b** our original knee-function score, **c** MOCART score. **p < 0.01 ***p < 0.001 (vs. PP)

Both the LKS and original knee-function score improved significantly from PP to EP I. There was no significant difference between the scores in EP I and EP II, indicating that this procedure lasted until approximately 6 years after implantation, although some patients showed deterioration of Lysholm and original knee scores between 1 year post implantation and the final follow-up (Table 4; Fig. 1).

**Table 4:** Clinical outcome scores and MRI findings for each patient

	Lysholm knee score			Original knee-function score			MRI score		
Case	PP	EP I	EP II	PP	EP I	EP II	PP	EP I	EP II
1	54	96	80	51	100	66	30	80	75
2	79	96	100	61	100	100	15	35	95
3	81	95	86	66	100	100	20	75	85
4	73	91	86	61	100	100	5	70	90
5	65	95	95	61	100	100	5	75	75
6	43	58	80	51	85	75	5	15	15
7	58	81	90	70	100	90	10	85	70
8	64	74	90	60	75	85	5	75	80
9	64	71	94	51	76	75	10	55	75
10	61	91	96	61	100	100	5	80	80
11	65	85	90	60	91	100	5	10	75
12	52	91	85	60	100	76	20	75	25
13	62	85	81	66	91	91	5	65	70
14	61	100	90	60	100	100	45	80	80

*PP* pre-implantation, *EP* *I* at 1 year post-implantation, *EP II* at the final evaluation time (EP II)

### Evaluation by MRI findings

#### MOCART scores

The mean (±SD) MOCART score was 13.2 (±12.03) pre-implantation, 62.5 (±24.71) at EP I, and 70.7 (±22.69) at EP II (Table 4, Fig. 1). The MOCART scores at EP I and EP II were significantly higher than the PP scores, but there was no significant difference between the scores at EP I and EP II.

We also evaluated various factors, including age, BMI, and disease of the patient, site and size of the lesions, and duration of the symptoms, which could be related to the final clinical scores and MOCART scores. However, we could not find any significant correlation between the clinical or MRI scores and the factors listed above, probably due to the small number of patients (data not shown).

### Case reports

#### Case no. 2: a male aged 26 years (at implantation), height 173 cm and weight 74.0 kg

**Fig. 2:**
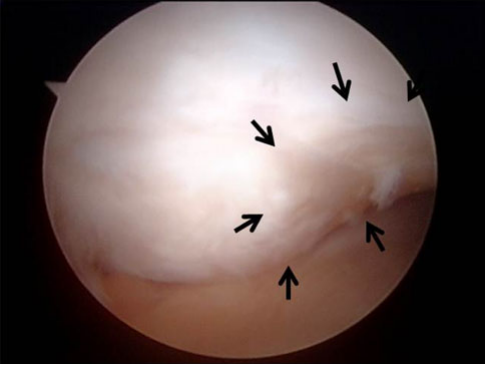
Case no. 2, a 26-year-old male. Arthroscopic view showing a 2.0 cm diameter traumatic cartilage defect in the lateral condyle of the left femur, classified as Outerbridge grade III

**Fig. 3:**
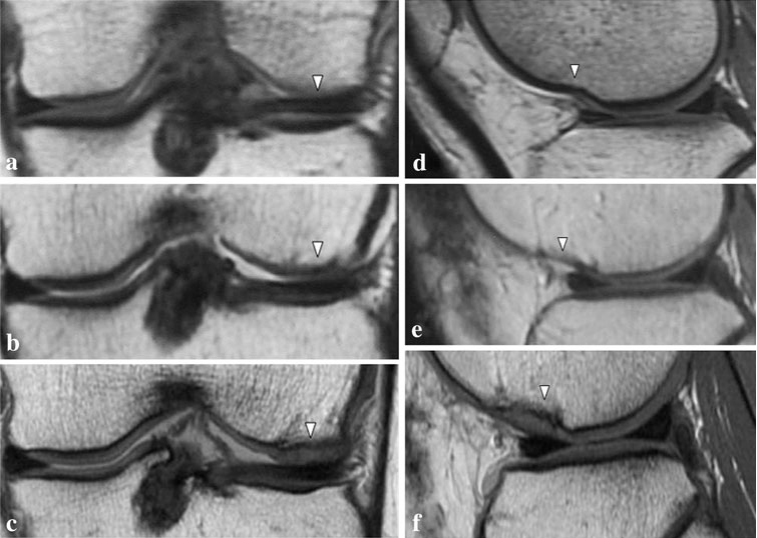
Case no. 2. There were cartilage defects (*arrowhead*) in the lateral condyle of the left femur (**a**, **d**). One year after operation, the thickness of the graft area was less than 50 % of that of normal cartilage (*arrowhead*) (**b**, **e**). At 6.6 years post-implantation, the signal intensity at the implanted site was almost the same (*arrowhead*) as that of surrounding normal cartilage (**c**, **f**)

Two months before implantation, this patient was diagnosed with a traumatic cartilage defect of the lateral condyle of the left femur. The LKS at PP was 79 points, and the original knee-function score was 61 points. MRI and arthroscopic examinations disclosed a 2.0 cm diameter cartilage defect in the lateral condyle of the left femur, classified as Outerbridge grade III (Fig. 2). The MOCART score at PP was 15 (Fig. 3a, d). Four weeks before implantation, cartilage tissue was harvested under arthroscopic guidance from a non-load-bearing region of the left patellofemoral cartilage and then used to prepare tissue-engineered cartilage, which was implanted into the cartilage defect in the patient’s left knee and then covered with periosteum harvested from the right tibia. For rehabilitation, CPM was started at 10 days post-implantation, partial weight-bearing flexion motion at 31 days post-implantation, and full weight-bearing flexion motion at 61 days post-implantation.

At EP I, the LKS was 96 points, and the original knee-function score was 100 points. MRI scans showed that tissue at the implanted site had slightly higher signal intensity than that of normal cartilage. The thickness was less than 50 % that of normal cartilage, and the MOCART score was 35 (Fig. 3b, e).

At 6.6 years post-implantation (EP II), the LKS was 100 points, and the original knee-function score was 100 points. The signal intensity at the implanted site was almost the same as that of surrounding normal cartilage except for a small low-intensity spot, and the implant thickness was almost the same as that of the surrounding normal cartilage. Although slight subchondral change is seen beneath the implanted site, the MOCART score was 95 (Fig. 3c, f).

#### Case no. 13: a female aged 52 years (at implantation), height 162 cm and weight 82.0 kg

**Fig. 4:**
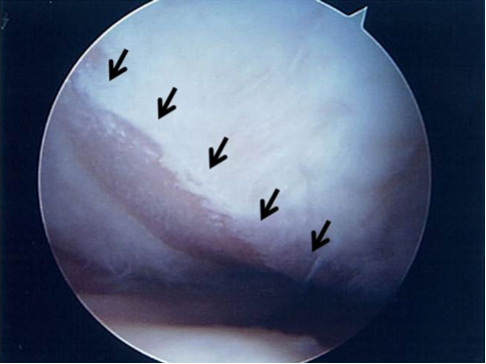
Case no. 13, a 52-year-old female. Arthroscopic view showing a 4.8 × 3.0 cm cartilage defect in the left medial femoral condyle, classified as Outerbridge grade IV

**Fig. 5:**
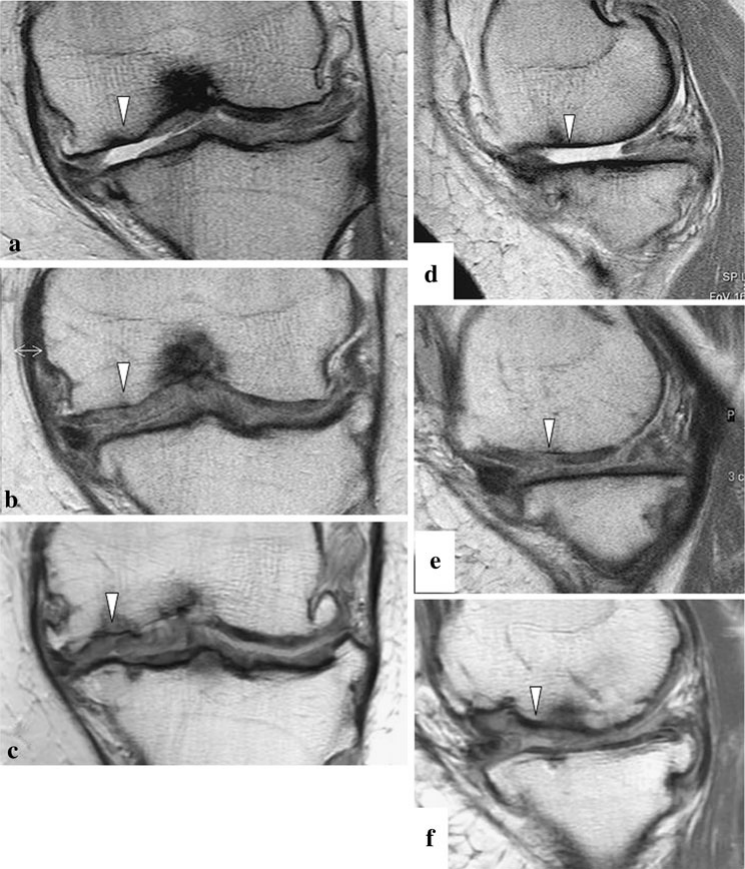
Case no. 13. An osteoarthritic cartilage defect occurred in the medial condyle of the left femur (*arrowhead*) (**a**, **d**). One year after operation, MRI showed that the thickness of the cartilage at the graft site (*arrowhead*) was almost the same as that of normal cartilage (**b**, **e**). At 5.7 years post-implantation, the thickness of the graft site (*arrowhead*) was maintained, although osteoarthritic change was slightly advanced (**c**, **f**)

Two years before implantation, the patient was diagnosed with osteoarthritis with cartilage defect in the left medial femoral condyle. The pre-implantation LKS was 62 points and the original knee-function score was 66 points. MRI and arthroscopic examinations disclosed a 4.8 × 3.0 cm cartilage defect in the left medial femoral condyle, classified as grade IV according to the Outerbridge scheme (Fig. 4). The MOCART score at PP was 5 (Fig. 5a, d).

At EP I, the LKS was 85 points and the original knee-function score was 91 points. MRI scans revealed that the cartilage defect was repaired with cartilaginous tissue with a signal intensity comparable to normal cartilage. The thickness of the cartilage at the site was almost the same as that of normal cartilage. The MOCART score was 65 (Fig. 5b, e).

At 5.7 years post-implantation (EP II), the LKS was 81 points and the original knee-function score was 91 points. MRI scans showed that the surface of the repaired tissue was slightly irregular and that the signal intensity was nonuniform, but the thickness was maintained until final follow-up, although osteoarthritic change was slightly advanced. The MOCART score was 70 (Fig. 5c, f). The femorotibial angles of this patient were 180° at PP, 176° at EP I, and 175° at EP II, indicating no further progression of deformity.

## Discussion

In this research, we obtained excellent results after implanting three-dimensionally cultured human chondrocytes grown in atelocollagen gel, which were prepared according to the method described by Ochi et al. To date, the findings have suggested that post-implantation formation of cartilage-like tissue is excellent, similar to the results obtained for autologous cartilage implantation (ACI) prepared in monolayer culture, as described by Brittberg et al*.* [1], although the histological findings of the repaired tissues were not examined in the present study to avoid damaging the repaired tissues. Furthermore, during our 6-year post-implantation evaluation period, no patients required further surgery. According to the comparative evaluation by Knutsen et al. [3], further surgery was required in 23 % of patients at 60 months after both ACI and microfracture treatment. During follow-up to 37 months described by Saris et al. [24], further surgery was required in 3.9 % of patients receiving characterized chondrocyte implants and 11.5 % of patients receiving microfracture surgery. Given these published results, it is clear that the results of long-term follow-up for our method were excellent, and compare favorably to those in previous reports on ACI.

By contrast, post-approval surveillance data for ACI in the United States show that adverse events were reported in 294 patients (497 events) in the period from 1996 to 2003. The most common adverse event was graft failure, reported in 73 patients (24.8 %), followed by delamination in 65 patients (22.1 %), tissue hypertrophy in 52 patients (17.7 %), and local infection in 21 patients (7.1 %) [5]. As there was no record of the total number of implantations, the incidence of adverse events cannot be calculated. Nevertheless, the adverse events that occurred were mainly associated with delamination or hypertrophy, and while the number of events was low, infection was also reported. In the report by Peterson et al. [21] describing research in which 94 patients were followed for 2–9 years after undergoing ACI, similar results were also obtained, with hypertrophy reported in 26 patients (27.6 %) and graft failure in 7 patients (7.4 %). In the earlier report of our research results, graft failure was reported in 2 of 31 subjects up to 1 year post-implantation. Of these 2 cases of graft failure, excessive flexion was forced in 1 case, and a hypertrophied portion became delaminated in the other. These findings were similar to many other case reports. Fortunately, transient pain was subsequently reported by only 2 patients up to the end of the present evaluation period, no patients required revision surgery during our follow-up, and no obvious adverse events were reported.

Usually, when evaluating treatment methods such as those described in this report, it is preferable to conduct a comparative study such as a randomized controlled trial (RCT). A number of reports describe efficacy evaluations of ACI made via RCTs [24, 25]. Unfortunately, for reasons associated with health insurance and other considerations, it would be difficult to conduct a complete RCT in Japan, and we were also unable to use such a design in the present clinical study. In brief, the reasons for not conducting an RCT included the following key ethical concerns. (1) Because implantation of tissue-engineered cartilage involves tissue being harvested for culture, it necessitates a different protocol from that of microfracture surgery alone, and the element of blinding is therefore lost. (2) Post-implantation follow-up would also require unnecessary arthroscopic examination to be conducted routinely in the control group of patients. (3) Patients who are seeking treatment with tissue-engineered cartilage would not be able to receive their desired treatment. (4) Minas et al. [26] reported problems with the prognosis for patients who were scheduled to undergo ACI if they had already received microfracture surgery beforehand. Hence, by using an MRI evaluation (given its relatively low level of invasiveness) in this study, we sought to ascertain the superiority of the treatment by observing changes from 1 to 6 years post-implantation, in addition to long-term follow-up for 6 years.

Quantifying joint cartilage defects or the state of their repair is difficult with MRI scans, and there have been almost no routinely established methods. To address this situation, Domayer et al. proposed a scoring system (MOCART score) that aimed to produce an objective evaluation using MRI scans [23]. Their method encompasses a comprehensive point score for examining MRI scans in post-implantation evaluation of repaired cartilage. This scoring system includes the height and signal intensity of the repaired cartilage, the state of integration with the surrounding tissue, the condition of the surface, and the state of formation of subchondral bone, which we believed was also the most appropriate indicator in our research. However, since an MRI imaging procedure for obtaining all of these evaluations was unavailable at the start of our study, we modified the method to comprise one type of imaging sequence to assess the signal intensity of the repair tissue.

As previously noted, our research is a comparison of the conditions at 1 year and 6 years post-implantation against that before implantation. Hence, as mentioned above, we were unable to use the results of our research to directly determine its superiority over other treatment methods. However, we were able to obtain findings that were not seen in earlier reports, such as temporal changes in MRI findings at each evaluation visit. The statistical analysis revealed no significant differences between the MRI findings at 1 year post-implantation and those at 6 years post-implantation, suggesting that the therapeutic benefit had been maintained. Although there were no statistically significant differences between the MOCART scores in EP I and EP II, we found a tendency for the scores to improve. Of course, we cannot draw any definitive conclusions from this study alone, and there is the possibility that this may be a characteristic of tissue-engineered cartilage implantation, because its condition after conventional cartilage repair procedure is anticipated to worsen. We definitely need more patients with a longer follow-up in the future to clarify this issue.

It could be claimed that the number of patients was not especially large and that the follow-up period was relatively short in the present study. Moreover, a retrospective approach was used for the MOCART score. Since the images required for the original method were unavailable, it was not possible to faithfully adhere to the original method, and hence a modified method was employed. To further improve the technique of implantation of tissue-engineered cartilage, it will now be necessary to accumulate more cases, and to conduct comparative analyses of variables such as MRI findings and various patient characteristics, severity of disease, and post-implantation management.

In our study, no patients required revision surgery after implantation, and the therapeutic outcomes were relatively stable from 1 year to about 6 years after implantation. These results could be attributed to the fact that the surgeons involved in the diagnosis and surgical procedure were well versed in this treatment method, and that the conditions of the operative procedure were adequately controlled. Moreover, the tissue-engineered cartilage used in our study was prepared at a rigorously controlled cell processing center, and was only supplied to the study after assuring quality via post-culture tests. To achieve clearance in relation to various biohazards and to minimize immunogenicity, a recommended rinsing step with residual serum was performed and a specified value for bovine serum albumin was imposed to assure safety. Moreover, standards for glycosaminoglycans (GAG) and type II collagen as well as the viable cell rate together with other variables were also used to assure efficacy. Under this control framework, the implanted tissue used in this research met all of the predetermined specification values. To stabilize the clinical outcomes achieved using this cultured cartilage in the future, it will be critically important to not only improve the surgical techniques and diagnostic capability of the surgeons and the safety of the cultured tissue used in implantation, but also to identify the efficacy parameters that can be used to establish performance.

In conclusion, although some patients showed deteriorations in their Lysholm and original knee scores between 1 year post-implantation and the final follow-up, we confirmed that the average clinical scores and MRI findings after implantation of tissue-engineered cartilage were improved at 1 year after implantation and were maintained until 6 years after implantation, indicating that our procedure has mid-term longevity.

## References

[1] Brittberg M, Lindahl A, Nilsson A, Ohlsson C, Isaksson O, Peterson L (1994). Treatment of deep cartilage defects in the knee with autologous chondrocyte transplantation. N Engl J Med.

[2] Knutsen G, Drogset JO, Engebretsen L, Grøntvedt T, Isaksen V, Ludvigsen TC, Roberts S, Solheim E, Strand T, Johansen O (2007). A randomized trial comparing autologous chondrocyte implantation with microfracture. Findings at five years. J Bone Joint Surg Am.

[3] Knutsen G, Engebretsen L, Ludvigsen TC, Drogset JO, Grøntvedt T, Solheim E, Strand T, Roberts S, Isaksen V, Johansen O (2004). Autologous chondrocyte implantation compared with microfracture in the knee. A randomized trial. J Bone Joint Surg Am.

[4] Wood JJ, Malek MA, Frassica FJ, Polder JA, Mohan AK, Bloom ET, Braun MM, Coté TR (2006). Autologous cultured chondrocytes: adverse events reported to the United States Food and Drug Administration. J Bone Joint Surg Am.

[5] Henderson I, Tuy B, Oakes B (2004). Reoperation after autologous chondrocyte implantation. Indications and findings. J Bone Joint Surg Br.

[6] Peterson L, Minas T, Brittberg M, Lindahl A (2003). Treatment of osteochondritis dissecans of the knee with autologous chondrocyte transplantation: results at two to ten years. J Bone Joint Surg Am.

[7] Peterson L, Vasiliadis HS, Brittberg M, Lindahl A (2010). Autologous chondrocyte implantation: a long-term follow-up. Am J Sports Med.

[8] Zaslav K, Cole B, Brewster R, DeBerardino T, Farr J, Fowler P, Nissen C (2009). A prospective study of autologous chondrocyte implantation in patients with failed prior treatment for articular cartilage defect of the knee: results of the study of the treatment of articular repair (STAR) clinical trial. Am J Sports Med.

[9] Browne JE, Anderson AF, Arciero R, Mandelbaum B, Moseley JB, Micheli LJ, Fu F, Erggelet C (2005). Clinical outcome of autologous chondrocyte implantation at 5 years in US subjects. Clin Orthop Relat Res.

[10] Krishnan SP, Skinner JA, Carrington RW, Flanagan AM, Briggs TW, Bentley G (2006). Collagen-covered autologous chondrocyte implantation for osteochondritis dissecans of the knee: two-to seven-year results. J Bone Joint Surg Br.

[11] Benya PD, Shaffer JD (1982). Dedifferentiated chondrocytes reexpress the differentiated collagen phenotype when cultured in agarose gels. Cell.

[12] Uchio Y, Ochi M, Matsusaki M, Kurioka H, Katsube K. Human chondrocyte proliferation and matrix synthesis cultured in atelocollagen gel. J Biomed Mater Res. 2000;50:138–43. 10.1002/(sici)1097-4636(200005)50:2<138::aid-jbm7>3.0.co;2-k 10679677

[13] Katsube K, Ochi M, Uchio Y, Maniwa S, Matsusaki M, Tobita M, Iwasa J. Repair of articular cartilage defects with cultured chondrocytes in atelocollagen gel. Comparison with cultured chondrocytes in suspension. Arch Orthop Trauma Surg. 2000;120:121–7. 10.1007/pl00021232 10738867

[14] Ochi M, Uchio Y, Kawasaki K, Wakitani S, Iwasa J (2002). Transplantation of cartilage-like tissue made by tissue engineering in the treatment of cartilage defects of the knee. J Bone Joint Surg Br.

[15] Frenkel SR, Di Cesare PE (2004). Scaffolds for articular cartilage repair. Ann Biomed Eng.

[16] Ossendorf C, Kaps C, Kreuz PC, Burmester GR, Sittinger M, Erggelet C (2007). Treatment of posttraumatic and focal osteoarthritic cartilage defects of the knee with autologous polymer-based three-dimensional chondrocyte grafts: 2-year clinical results. Arthritis Res Ther.

[17] Della Villa S, Kon E, Filardo G, Ricci M, Vincentelli F, Delcogliano M, Marcacci M. Dose intensive rehabilitation permit early return to sport without compromising the clinical outcome after arthroscopic autologous chondrocyte implantation in highly competitive athletes?. Am J Sports Med. 2010;38:68–77. 10.1177/0363546509348490 20051508

[18] Roos EM, Roos HP, Lohmander LS, Ekdahl C, Beynnon BD. Knee Injury and Osteoarthritis Outcome Score (KOOS)—development of a self-administered outcome measure. J Orthop Sports Phys Ther. 1998;28:88–96. 10.2519/jospt.1998.28.2.88 9699158

[19] Lysholm J, Gillquist J (1982). Evaluation of knee ligament surgery results with special emphasis on use of a scoring scale. Am J Sports Med.

[20] Tohyama H, Yasuda K, Minami A, Majima T, Iwasaki N, Muneta T, Sekiya I, Yagishita K, Takahashi S, Kurokouchi K, Uchio Y, Iwasa J, Deie M, Adachi N, Sugawara K, Ochi M (2009). Atelocollagen-associated autologous chondrocyte implantation for the repair of chondral defects of the knee: a prospective multicenter clinical trial in Japan. J Orthop Sci.

[21] Peterson L, Minas T, Brittberg M, Nilsson A, Sjögren-Jansson E, Lindahl A (2000). Two-to 9-year outcome after autologous chondrocyte transplantation of the knee. Clin Orthop Relat Res.

[22] Domayer SE, Welsch GH, Dorotka R, Mamisch TC, Marlovits S, Szomolanyi P, Trattnig S (2008). MRI monitoring of cartilage repair in the knee: a review. Semin Musculoskelet Radiol.

[23] Welsch GH, Mamisch TC, Quirbach S, Zak L, Marlovits S, Trattnig S (2009). Evaluation and comparison of cartilage repair tissue of the patella and medial femoral condyle by using morphological MRI and biochemical zonal T2 mapping. Eur Radiol.

[24] Saris DB, Vanlauwe J, Victor J, Almqvist KF, Verdonk R, Bellemans J, Luyten FP (2009). Treatment of symptomatic cartilage defects of the knee: characterized chondrocyte implantation results in better clinical outcome at 36 months in a randomized trial compared to microfracture. Am J Sports Med.

[25] Levine DW, Roaf PL, Duguay SJ (2009). Characterized chondrocyte implantation results in better structural repair when treating symptomatic cartilage defects of the knee in a randomized controlled trial versus microfracture. Am J Sports Med.

[26] Minas T, Gomoll AH, Rosenberger R, Royce RO, Bryant T (2009). Increased failure rate of autologous chondrocyte implantation after previous treatment with marrow stimulation techniques. Am J Sports Med.

